# Transcriptomic diversification of granulosa cells during follicular development between White Leghorn and Silky Fowl hens

**DOI:** 10.3389/fgene.2022.965414

**Published:** 2022-07-26

**Authors:** Yurong Tai, Xue Yang, Deping Han, Zihan Xu, Ganxian Cai, Jiaqi Hao, Bingjie Zhang, Xuemei Deng

**Affiliations:** ^1^ Key Laboratory of Animal Genetics, Breeding and Reproduction of the Ministry of Agriculture & Beijing Key Laboratory of Animal Genetic Improvement, China Agricultural University, Beijing, China; ^2^ College of Veterinary Medicine, China Agricultural University, Beijing, China; ^3^ Hainan Sanya Research Institute, Seed Laboratory, Sanya, China

**Keywords:** granulosa cell, transcriptome, White Leghorn, Silky Fowl, melanocyte

## Abstract

Egg production rate in chicken is related to the continuity of follicle development. In this study, we found that the numbers of white prehierarchical, dominant, and yellow preovulatory follicles in the high-yielding layer breed, White Leghorn (WL), were significantly higher than those in the low egg-yielding variety, Silky Fowl (SF). The proliferation and differentiation of granulosa cells (GCs) play an important role in follicle maturation. Histological observation revealed a large number of melanocytes in the outer granulosa layer of follicles in SF but not in WL. Finally, RNA-sequencing was used to analyze the gene expression profiles and pathways of the GC layer in the follicles in both WL and SF hens. Transcriptome analysis of prehierarchical GCs (phGCs) and preovulatory GCs (poGCs) between WL and SF showed that steroid hormone-, oxytocin synthesis-, tight junction-, and endocytosis-related genes were expressed at higher levels in WL phGCs than in SF phGCs, whereas the insulin signaling pathway- and vascular smooth muscle contraction-related genes were upregulated in SF phGCs. Fatty acid synthesis, calcium signaling, and Wnt signaling pathway-related genes were expressed at higher levels in WL poGCs than in SF poGCs; however, adrenergic signaling, cGMP-PKG, and melanogenesis-related genes were upregulated in SF poGCs. These results indicate that genes that promote GC proliferation and secretion of various sex hormones are more active in WL than in SF hens. The upregulated signaling pathways in SF help in providing energy to GCs and for angiogenesis and melanogenesis. *In vitro* experiments confirmed that both the proliferation of poGCs and synthesis of reproductive hormones were higher in WL than in SF hens.

## Introduction

The growth, development, and function of follicles determine the egg-laying performance of chickens (Li et al., 2019). Mature hens have numerous ovarian follicles. According to their different developmental stages, the follicles can be roughly divided into prehierarchical follicles (ph fc) and preovulatory follicles (po fc) (Johnson, 2015; Wang and Gong, 2017). The existing theory of follicular development in chickens holds that every time an ovulation event occurs, ph fc enters follicle selection. Selected dominant follicle undergoes rapid development from F5 to F1 follicle stages until ovulation (Johnson and Woods, 2009). Therefore, the transition from ph fc to po fc is considered very important and may determine the egg-laying performance of hens.

The follicle structure from the center to the periphery consists of a single yolk-filled oocyte germ cell, perivitelline cells (PVL), granulosa cells (GCs), basement membrane, and theca cells (Schmierer et al., 2003). The proliferation and differentiation of GCs play a decisive role in follicular development. GC differentiation regulates the initiation of primordial follicles. Studies have shown that the stem cell factor (SCF) expressed and secreted after GC differentiation can activate the development of primordial follicles (Zhang et al., 2014). In poultry, the apoptosis of GCs mainly plays a role in the atresia of growing follicles, whereas GCs of po fc have strong anti-apoptotic ability (Johnson, 2015). GC differentiation is involved in follicle selection. Elevated follicle stimulating hormone receptor (FSHR) and cyclic adenosine monophosphate (cAMP) levels in GCs are widely recognized as markers of follicle selection (Johnson, 2015). Therefore, research into the differences in phGC and poGC gene expression between high-yield and low-yield hens will help us understand the reasons for the differences in their laying performance.

White Leghorn (WL) is a representative high-laying breed native to Italy. It is one of the main parents of modern, high egg-yielding hens. Silky Fowl (SF) is a representative breed of black bone chicken, native to China. The growth curve and body shape of SF and WL are similar, but the egg-laying performance of SF is much lower than that of WL. SF stems from a natural mutation, one of which is thought to be the inversion and duplication of endothelin 3 (*EDN3*) (Han et al., 2015). In addition to the pigmented skin and internal organs in the SF breed, the ovaries are also covered with melanin, which may affect their reproductive ability. In this study, RNA-sequencing was used to identify the differences in gene expression and their signaling pathways in the GC tissue at different stages of follicular development in high- and low-yield hens to understand the molecular regulatory mechanisms of their egg-laying performance.

## Materials and methods

### Collection of chicken follicles and granulosa cells tissue

Healthy, 240-day-old laying hens (WL and SF) were provided by the China Agricultural University Poultry Genetic Resources and Breeding Test Base (Beijing, China). In this study, 150 each of WL and SF chickens were selected for observation and sampling. They were raised in the same house with free feeding and drinking. According to the needs of the experiment, three WLs and three SFs at 30 weeks were selected to collect ph fc with diameter of 6‒8 mm and po fc with diameter of 9–40 mm. The hens were euthanized by cervical dislocation before GC layer collection for transcriptome sequencing.

Animals were reared and handled in accordance with the “Guidelines for the Care and Use of Laboratory Animals in China” and in compliance with the Beijing Municipal Laboratory Animal Welfare and Ethics Guidelines. All experimental protocols were approved by the Animal Experiment Ethics Committee of China Agricultural University (license number: SKLAB-2012-04-07).

### Harvesting of follicle granulosa cells layers and RNA extraction

RNA extraction from the GC layer of the follicle was performed by removing the follicle from the ovary (Gilbert et al., 1977) and adding it to 4 °C DPBS (Gibco-BRL, Grand Island, NY, United States). The yolk was released and rinsed with DPBS until no obvious yolk remained. Total RNA was extracted using TRIzol reagent (Invitrogen, Carlsbad, CA, United States) following the manufacturer’s instructions.

### RNA sequencing and data analysis

RNA purity and quantification were evaluated using the NanoDrop 2000 spectrophotometer (Thermo Fisher Scientific, former Savant, MA, United States). RNA integrity was assessed using the Agilent 2100 Bioanalyzer (Agilent Technologies, Santa Clara, CA, United States). Then the libraries were constructed using TruSeq Stranded mRNA LT Sample Prep Kit (Illumina, SD, California, United States) according to the manufacturer’s instructions. The transcriptome sequencing and analysis were conducted by OE Biotech Co., Ltd. (Shanghai, China).

The libraries were sequenced on an Illumina HiSeq X Ten platform and 150 bp paired-end reads were generated. About 17156 raw reads for each sample were generated. Raw reads of fastq format were firstly processed using Trimmomatic (Bolger et al., 2014) and the low quality reads were removed. Then about 653 clean reads for each sample were retained for subsequent analyses. The clean reads were mapped to the gallus genome website (ftp://ftp.ensembl.org/pub/release-74/fasta/gallus_gallus/dna/) using HISAT2 (Kim et al., 2015). Fragments per kilobase of exon model per million mapped fragments (FPKM) of each gene was calculated using Cufflinks (Trapnell et al., 2012), and the read counts of each gene were obtained by HTSeqcount. Differential expression analysis was performed using the DESeq (2012) R package. *p* value <0.05 and fold change >2 or <0.5 was set as the threshold for significant differential expression. Hierarchical cluster analysis of differentially expressed genes (DEGs) was performed to demonstrate the expression pattern of genes in different groups and samples. Gene Ontology (GO) and Kyoto Encyclopedia of Genes and Genomes (KEGG) pathway enrichment analysis of DEGs were performed respectively using R based on the hypergeometric distribution.

### Quantitative real-time PCR validation

Total RNA isolated from follicle granulosa tissue were extracted by using Invitrogen TRIzol reagent (Invitrogen, Carlsbad, CA, United States), following the manufacturer’s instructions. A total of 1 μg RNA was reverse transcribed into cDNA using the Fast Quant RT Kit (with gDNase) (Tiangen Biotech Co., Ltd., Beijing, China) according to manufacturer’s instructions. The expression levels of *WNT4*, *LHCGR*, *HSD11B2*, *EDN3*, *EDNRB2*, *ADIPOQ*, *CD38*, *STAR*, *HSD3B1*, *CYP11A1*, *FSHR*, and *ER* were quantified by qPCR using the SYBR Green Real-time PCR Master Mix (Tiangen Biotech Co., Ltd., Beijing, China). The primers for qPCR were designed using the Primer Premier 5 software (PREMIER Biosoft, Palo Alto, CA) and were subsequently synthesized (Sangon Biotech Co., Ltd., Beijing, China). The primer information is listed in Table 1. The cycling parameters used for qPCR amplification were as follows: initial heat-denaturation at 95°C for 4 min; 40 cycles of 95°C for 30 s, 55–60°C for 30 s, and 72°C for 30 s; and a final extension at 72°C for 5 min. A melting curve analysis was performed to exclude genomic DNA contamination and to confirm primer specificities. Gene expression was normalized using the 2^−△△CT^ method with the glyceraldehyde 3-phosphate dehydrogenase (*GAPDH*) gene as an internal standard. Each biological duplicate was controlled in three technical replicates.

**TABLE 1:** The primers used to detect the targeted genes by qPCR.

Genes	Primer sequence (5′-3′)	Annealing temperature (°C)	Size (bp)
WNT4	F-CGAGCTGGACAAGTGTGGAT R-GGACGTCGACAAAGGACTGT	60	125
LHCGR	F-ACGAATCGCTGACACTCAAAC R-CTCTCAGGGCATCGTTGTGT	60	138
HSD11B2	F-CACACCAATGGCACAGGTCTC R-GTGCGGAAGTTGCCCAATG	61	98
EDN3	F-TCAACACCCCAGAGAGGACT R-AGAGCACCGAAATGAAGGCT	59	116
EDNRB2	F-CCTCATTGCTCTGCCCATCA R-GCCACTGCTCGATACCTGTC	58	159
ADIPOQ	F- GAAGGGACGGCAAGG R- TCACCCGTGTCCCCT	56	85
CD38	F-AGACAATTACAGGCCAGTCC R-CATGTCCTTTTCAGTTCTGCAC	57	152
STAR	F-CCATCAGCCAGGAGCTCAG R-ATCTCGCTGAAGGGCTTCTC	58	124
HSD3B1	F-GCCAGAGGATCGTTCGCTTA R-CATCTCGGATGTCCCCTTCC	59	153
CYP11A1	F-TACCGTGACTACCGCAACAA R-AAAAAGTCCTGGCTCACCTGG	60	152
FSHR	F-GAGCGAGGTCTACATACA R-GCACAAGCCATAGTCA	55	281
ER	F-TATTGATGATCGGCTTAGTCTGGC R-CGAGCAGCAGTAGCCAGTAGCA	63	145
GAPDH	F-TCGGAGTCAACGGATTTGGC R-ACAGTGCCCTTGAAGTGTCC	60	163

### Granulosa cells culture

GC layers were isolated from poGCs (mixed F1-F6 follicles) according to a previously reported method (Gilbert et al., 1977). The specific process is to peel off the connective tissue of the follicle surface, discard the yolk, and carefully separate the membranous and the GC layers from the follicle. The GC layer was collected in a dish and rinsed twice with DPBS. The GC layer was chopped and digested with 0.2% collagenase II (Gibco) at 37°C for 10–15 min. The digestion was terminated by adding M199 (Gibco) medium containing 10% fetal bovine serum, filtered and centrifuged. Cell pellets were taken and cultured in an incubator at 37°C and 5% CO_2_.

### Cell viability assay

GCs viability was determined using Cell counting kit-8 (CCK-8) assay. GC culture medium (0.1 ml; 1 × 10^5^ cells/mL) were plated in 96-well plates and were grown in M199 medium overnight. CCK-8 (10 μL; Beyotime, Nanjing, China) was added into each well and incubated for 2 h. Finally, optical density was measured at 450 nm of each well using a spectrophotometer (Bio-Rad Laboratories, Hercules, CA, United States).

### Enzyme-linked immunosorbent assay

The culture supernatant was collected from the cultured cell wells and centrifuged gently; the supernatant was used for further experiments. The levels of follicle-stimulating hormone (FSH), luteinizing hormone (LH) and progesterone (P4) secreted by GCs in the culture supernatant were detected by ELISA kit (Shanghai Enzyme-linked Biotechnology Co., Ltd., Shanghai, China) according to the manufacturer’s instructions. Standards and samples were added to ELISA plate wells. The horseradish peroxidase (HRP) labeled antigen and detection antibody were added first, followed by the reaction mixture. The plate was washed after 60 min of incubation at 37°C. The enzyme-linked working solution was added, and the mixture was incubated for 15 min. The reaction was terminated with a stop solution, and the OD_450_ value was finally determined.

### Statistical analyses

All statistical analyses were performed using Prism 6.0 (GraphPad Software). Data are expressed as means ± standard error of the mean (SEM). Statistical significance was evaluated using Student’s t test. Asterisk coding is indicated in the Figure Legends as *p < 0.05; **p < 0.01; ***p < 0.001; ****p < 0.0001.

## Results

### Difference in egg production between White Leghorn and Silky Fowl hens is associated with the number of follicles

WL and SF hens were raised in similar caged environments, and the egg production rate from weeks 20–59 was recorded and calculated weekly (Supplementary Additional File S1). WL egg production rates increased rapidly from the start of laying, the peak lasted for approximately 10 weeks, and the decline after the peak was slow. SF egg production rates increased slowly after the start of laying and the duration of the peak was only approximately 4 weeks. After this peak, the egg-laying rate decreased rapidly. The egg-production rate curves showed that with an increase in days, the difference between the egg-laying rates of SF and WL also increased.

**FIGURE 1:**
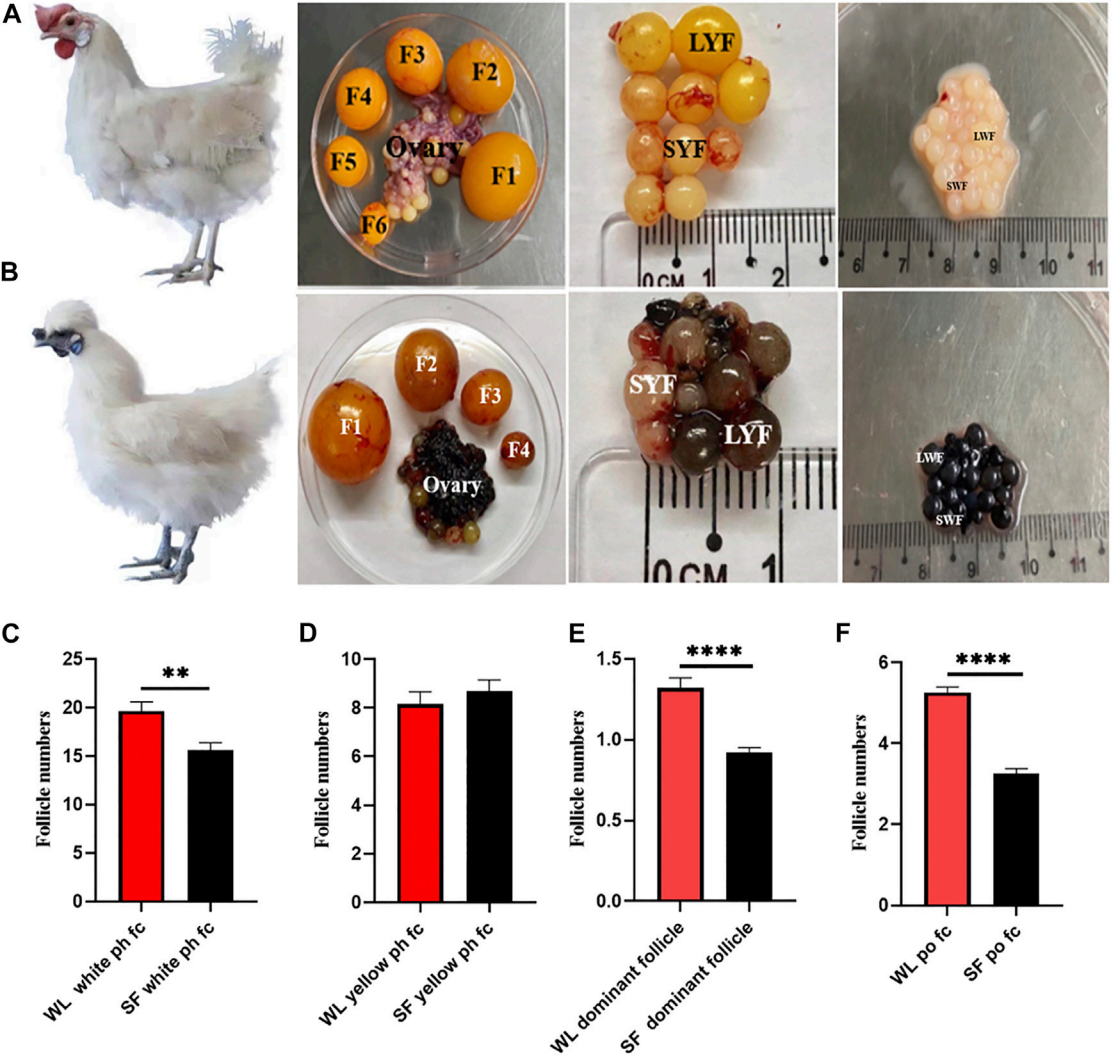
Follicular morphology of WL and SF. **(A)** Morphology of WL prehierarchical and preovulatory follicles. **(B)** Morphology of SF prehierarchical and preovulatory follicles. (SWF, small white follicle; LWF, large white follicle; SYF, small yellow follicle; LYF, large yellow follicle). **(C)** Compare the number of WL and SF white prehierarchical follicles. **(D)** Compare the number of WL and SF yellow prehierarchical follicles. **(E)** Compare the number of WL and SF dominant follicles. **(F)** Compare the number of WL and SF preovulatory follicles.

We compared the number of follicles at different developmental stages in both WL and SF ovaries. The number of white ph fc in WL was significantly higher than that in SF (Figure 1C), whereas the number of yellow ph fc did not differ significantly (Figure 1D). The number of dominant follicles in WL ovaries was significantly higher than that in SF (Figure 1E). The number of po fc in WL was significantly higher than that in SF (Figure 1F). The results showed that the most obvious difference between WL and SF was the number of white prehierarchical, dominant, and preovulatory follicles. These results also indicated that differences in follicular development have an important impact on egg production continuity and egg production in both breeds.

### Differentially expressed genes in White Leghorn and Silky Fowl phGCs

**FIGURE 2:**
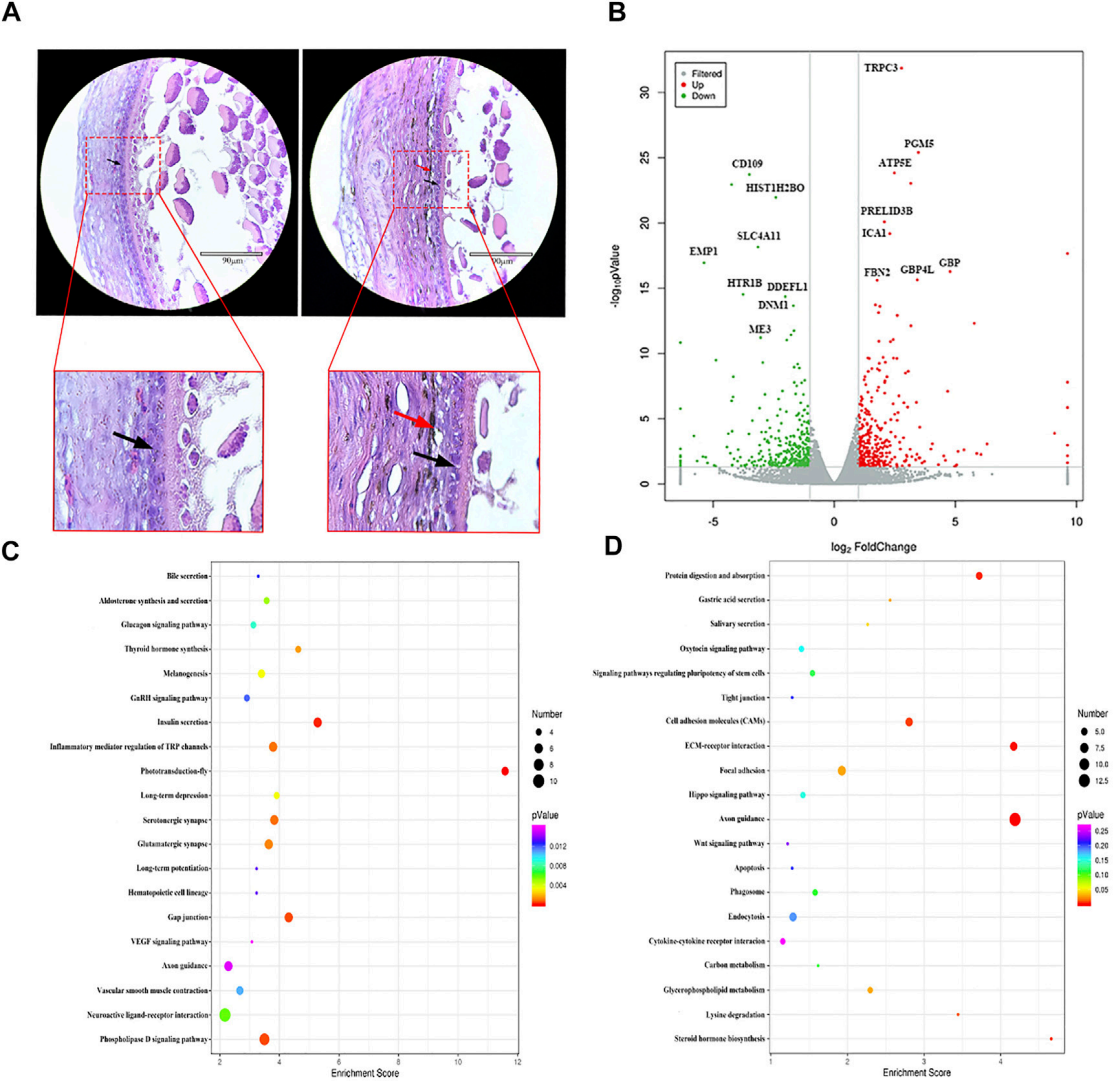
Comparison of gene expression differences between WL and SF phGCs. **(A)** Histological observation of WL and SF phGCs. (Left is WL, right is SF. black arrow, follicle granulosa cells; red arrow, melanocytes). **(B)** Volcano plot of up- and down-regulated genes. **(C)** Top 20 KEGG pathway of up-regulated genes in SF phGCs compared with WL phGCs. **(D)** Top 20 KEGG pathway of down-regulated genes in SF phGCs compared with WL phGCs. The x-axis is the enrichment score, the larger the bubble, the greater the number of genes encoding differential proteins. The color of the dots represents the range of q values.

GCs are the most important structures in follicles, and their growth and differentiation determine the same in follicles (Johnson, 2015). Histological observation results showed that WL and SF ph fc were double layered and densely arranged. Compared with that in WL, a large number of melanocytes and melanin were distributed outside the SF phGC layer (Figure 2A).

We selected WL and SF ph fc with a diameter of approximately 6 mm to obtain GC layer tissues for high-throughput RNA sequencing. There were 653 DEGs (according to FC > 2 and *p* value < 0.05); 315 genes were upregulated and 338 genes were downregulated in SF (Figure 2B). Through the analysis and classification of phGC DEGs, it was found that the expression of genes related to cell growth and development was significantly increased in WL. For example, the regulator of cell cycle gene (*RGCC*), *WNT2*, a member of the WNT family, was involved in cell growth. In addition, genes related to sex hormone synthesis and secretion were also found to be expressed at high levels, including fork head box protein 1 (*FOXA1*), which is involved in estrogen receptor-mediated transcription and plays an important role in promoting follicular selection and differentiation. The genes upregulated in SF included interleukin 7 receptor (*IL7R*), which is related to immunity; transmembrane protein 156 (*TMEM156*), which is related to substance transport; and *EDN3* which is related to melanocyte formation and migration.

To reveal the relationship between these genes and known biological processes, cellular components, and molecular functions, the GO terms of the significant enrichment of DEGs were summarized. A total of 23 GO terms were categorized into various biological processes, including growth, reproduction, and developmental processes. A total of 20 GO terms were categorized into cellular components, including cell junctions, membranes, and nucleoids. Another 21 GO terms were categorized into molecular function, such as, molecular transducer activity, protein-binding transcription factor activity, and translation regulator activity (Supplementary Additional File S2).

KEGG pathway analysis showed that among the top 20 signaling pathways, using a threshold q-value <0.05, the upregulated gene pathways in SF included insulin secretion, thyroid hormone synthesis, melanin production, and the vascular smooth muscle contraction signaling pathways (Figure 2C). Downregulated gene pathways in SF included steroid hormone biosynthesis, the oxytocin signaling pathway, endocytosis, and the Wnt signaling pathway (Figure 2D).

These results suggest that the activated genes and pathways in WL phGCs are involved in the promotion of follicular growth and development. In addition, they are also related to the selection of follicles as dominant follicles and subsequent important biological events in follicle development, such as upregulation of genes related to the synthesis of steroid hormones and oxytocin. In contrast, genes with high expression in SF include that of insulin and thyroxine synthesis related to energy supply, as well as genes related to melanocyte synthesis and their own biological characteristics, which correspond to the histological observation of follicles.

### Differentially expressed genes in White Leghorn and Silky Fowl poGCs

**FIGURE 3:**
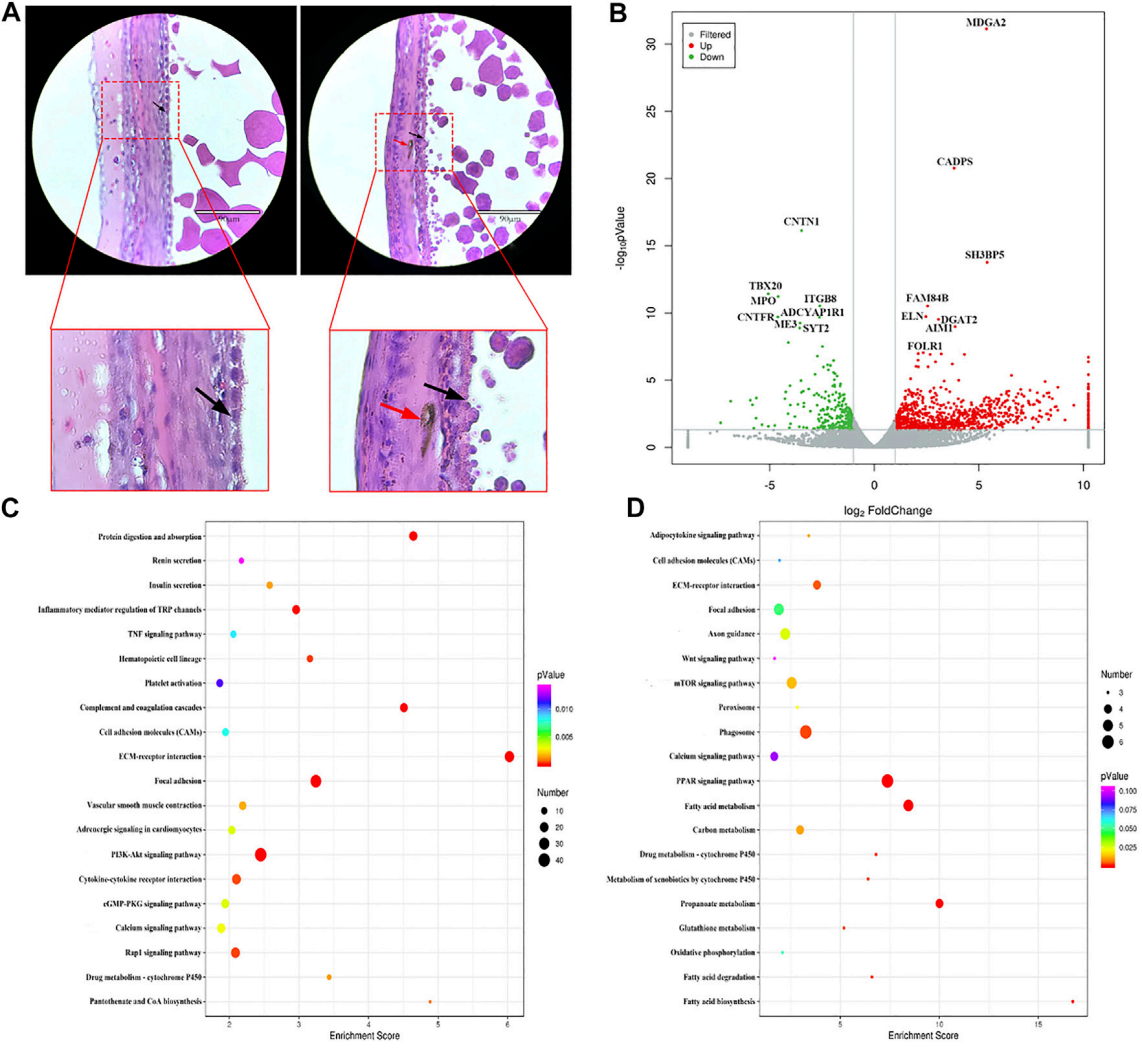
Comparison of gene expression differences between WL and SF poGCs. **(A)** Histological observation of WL and SF poGCs. (Left is WL, right is SF. black arrow, follicle granulosa cells; red arrow, melanocytes). **(B)** Volcano plot of up- and down-regulated genes. **(C)** Top 20 KEGG pathway of up-regulated genes in SF poGCs compared with WL poGCs. **(D)** Top 20 KEGG pathway of down-regulated genes in SF poGCs compared with WL poGCs. The x-axis is the enrichment score, the larger the bubble, the greater the number of genes encoding differential proteins. The color of the dots represents the range of q values.

Histological observation of the WL and SF poGC layers revealed a change from a double-layer (phGC layer) to a single-layer structure. Compared with that in WL, there were still a large number of melanocytes near the GC layer of SF (Figure 3A). We selected WL and SF poGCs with a diameter of approximately 9–40 mm follicle to obtain GC-layer tissues for high-throughput RNA sequencing. There were 1081 DEGs (FC > 2 and *p* < 0.05). Among them, 822 were upregulated and 259 were downregulated in SF poGCs compared to that in WL poGCs (Figure 3B). The top 50 genes that were highly expressed in WL included phosphodiesterase 1B (*PDE1B*), immunoglobulin superfamily member 10 (*IGSF10*), and troponin I1 (*TNNI1*). The top 50 genes that are highly expressed in SF include the apolipoprotein L domain (*APOLD1*), hydroxysteroid 17-beta dehydrogenase 2 (*HSD17B2*), adiponectin, C1Q and collagen domain containing (*ADIPOQ*).

To reveal the functional aspects of these DEGs, 64 GO terms were summarized for biological processes, cellular components, and molecular functions. A total of 23 GO terms were categorized as biological processes, including cellular processes, regulation of biological processes, and rhythmic processes. 20 were categorized into cellular components, including the extracellular matrix, membrane, and virion; and 21 were categorized into molecular functions, including electron carrier activity, nucleic acid binding, transcription factor activity, and receptor activity (Supplementary Additional File S3).

Among the top 20 KEGG enrichment-signaling pathways, the upregulated gene pathways in SF poGCs included insulin secretion, cGMP-PKG, TNF, and vascular smooth muscle contraction (Figure 3C). The gene pathways down regulated in SF poGCs included fatty acid biosynthesis, phagosomes, cell adhesion molecules, and the Wnt signaling pathway (Figure 3D). We also focused on other signaling pathways related to GC growth, development, and cell-cell interactions, although they did not belong to the top 20 categories. The upregulated signaling pathways in WL included ovarian steroidogenesis, steroid hormone biosynthesis, and the oxytocin-signaling pathway. The upregulated signaling pathways in SF included aldosterone synthesis and secretion, the MAPK signaling pathway, and the melanogenesis pathway.

These results showed that the ability of WL poGCs to synthesize and secrete ovarian steroid hormones was significantly higher than that of SF. The synthesis and secretion of steroid hormones is indispensable for follicular development in the po fc stage and also during ovulation (Cecconi et al., 2004). The expression of genes related to sex hormone synthesis in SF poGCs was significantly lower than that in WL, but the ability to communicate with information and substances was higher. A large number of genes related to melanocyte synthesis were screened in SF, which was consistent with our histological observation.

### Validation of differential gene expression in phGCs and poGCs

**FIGURE 4:**
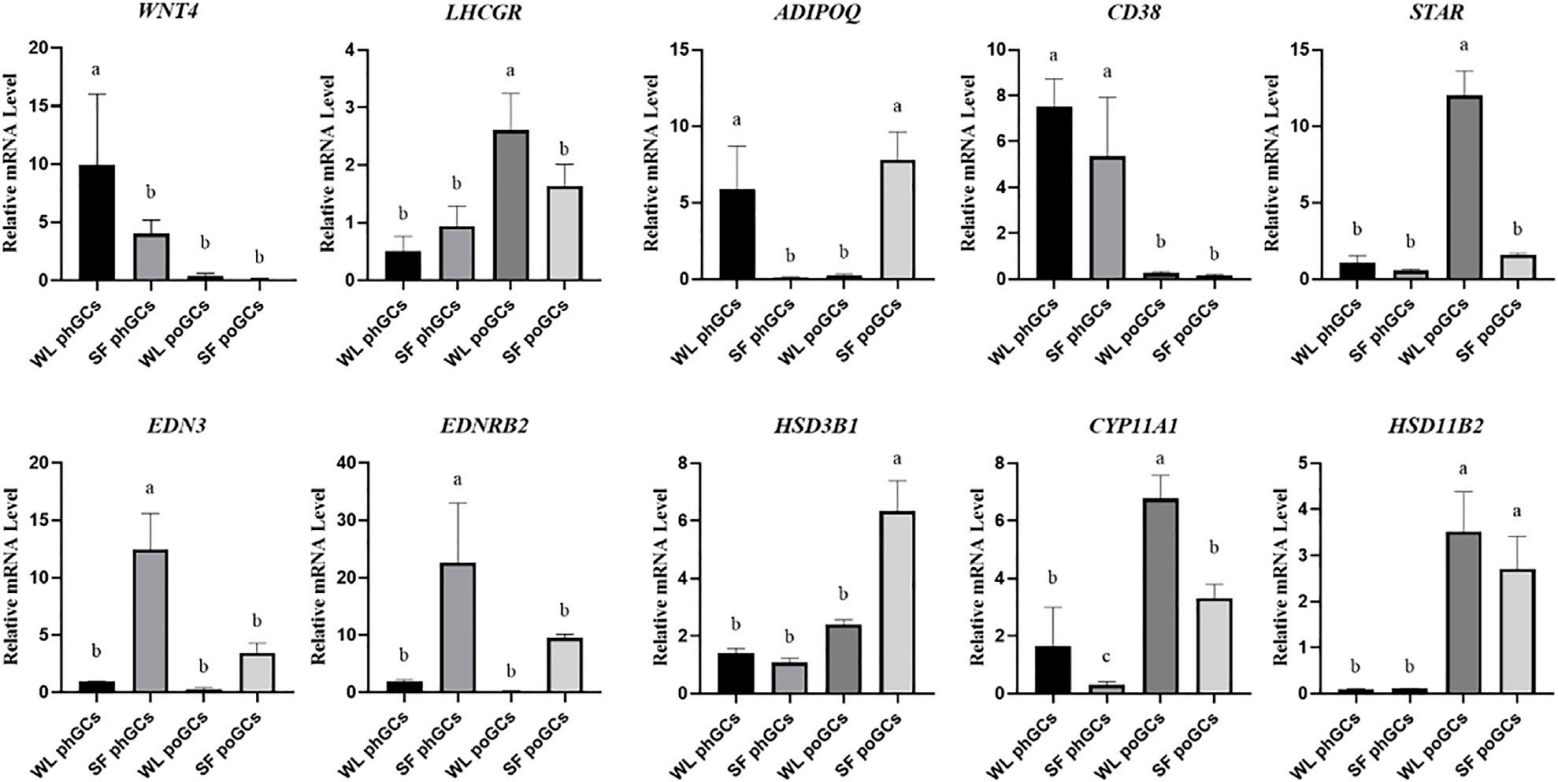
Quantitative real-time PCR validation of differentially expressed genes identifed in transcriptome sequencing.

To confirm the reliability of gene expression profiles obtained using high-throughput RNA sequencing, real-time quantitative PCR analysis was performed. In the two developmental stages, genes related to steroid synthesis (*LHCGR*, *STAR*, *HSD3B1*, *CYP11A1*, and *HSD11B2*), germ cell development factor (*WNT4*), transmembrane glycoprotein-encoding gene (*CD38*), and promote hormone metabolism during blood circulation gene (*ADIPOQ*) were used as validation genes. The qPCR results were consistent with the significant differences observed in the high-throughput sequencing data (Figure 4).

### *In vitro* proliferation and hormone synthesizing ability of White Leghorn and Silky Fowl poGCs

**FIGURE 5:**
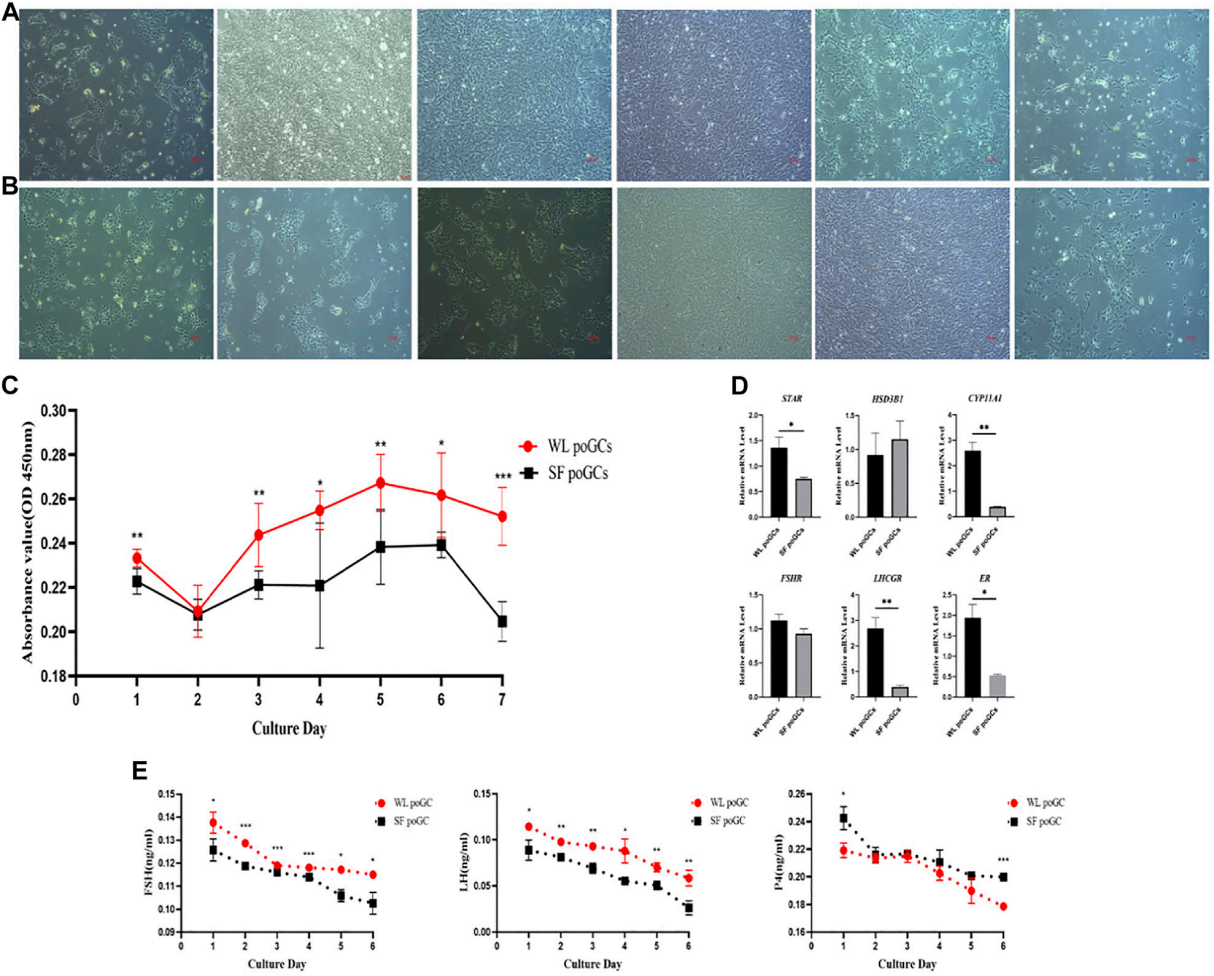
Comparison of proliferation and differentiation between WL and SF poGCs cultured *in vitro*. **(A)** Morphology of WL poGCs cultured *in vitro* (1–6 days). **(B)** Morphology of SF poGCs cultured *in vitro* (1–6 days). **(C)** Comparison of WL poGCs and SF poGCs cell proliferation ability. **(D)** Expression of genes associated with the synthesis of sex hormones and receptors in GCs were detected. **(E)** Comparison of secretion FSH, LH and P4 between WL poGCs and SF poGCs. (*p < 0.05; **p < 0.01; ***p < 0.001).

To explore the difference in biological function between high- and low-yield layered GCs, we isolated and cultured WL and SF poGCs from 1 to 6 days *in vitro* (Figures 5A,B). We detected the proliferative ability of primary isolated and cultured poGCs from WL and SF using the CCK8 method. The proliferation ability of WL poGCs gradually increased from day 2–5 and decreased from day 5–7. The proliferation ability of SF decreased rapidly from day 6 onwards. The proliferation capacity of WL poGCs was significantly higher than that of SF (Figure 5C).

The synthesis and secretion of sex hormones by GCs is important for follicular maturation and ovulation. We examined the differences in the expression of related hormone and receptor genes between WL and SF poGCs. The expression levels of *STAR*, *CYP11A1*, *LHCGR*, and *ER* in WL poGCs were significantly higher than those in SF poGCs; however, there was no significant difference in the expression levels of *HSD3B1* and *FSHR* between the two groups (Figure 5D).

We also measured the changes in sex hormone synthesis in poGCs from the two breeds using ELISA for FSH, LH, and P4. The results showed that hormone secretion from the primary GCs in the two breeds decreased gradually within 1–6 days of *in vitro* culture. Except for P4, the secreted levels of FSH and LH were higher in WL than in SF (Figure 5E). The *in vitro* results showed that WL poGCs exhibited stronger cell proliferation and hormone synthesis than SF poGCs.

## Discussion

Egg production is related to the number and development of ovarian follicles (Hlokoe et al., 2022). Our results showed that the number of white prehierarchical, dominant, and preovulatory follicles in high-yielding hens was significantly higher than that in low-yielding ones (Figures 1A–F). White prehierarchical follicles, which serve as the follicle pool, are an important reserve for the sustainable development of follicles and are also very important for dominant follicle selection (Chen Q. et al., 2020). WL has a larger follicle pool than SF. The formation of dominant follicles is an most important stage in follicular development, and these follicles have highest energy and strongest hormone synthesizing ability compared with that of the other follicles (Wang et al., 2017). High-yielding hens usually have 1–2 dominant follicles at peak laying, whereas the number of dominant follicles of low-yielding hens is 0–1. The difference in the number of dominant follicles leads to a decrease in the po fc number and disruption of developmental continuity. The number of po fc directly affects the continuity of ovulation (Brady et al., 2021), and the continuity of follicular development determines the egg-laying performance in chickens (Evans, 2003). We found that the po fc in WL had six grades (F1‒F6), whereas follicles in SF mostly had four grades (F1‒F4). The difference in the number of follicles in the ph fc, dominant follicle, and po fc is the direct reason why the egg-laying rate of WL is higher than that of SF.

In birds, follicle maturation is closely related to the functional differentiation of the GC layer (Woods and Johnson, 2005). While the oocytes of the primary follicles begin to divide and mature, the follicles continue to grow and develop into secondary follicles, and the GCs proliferate rapidly from a single-layered to a multi-layered structure. Secondary follicles develop further, accumulate yolk material, and gradually protrude from the surface of the ovarian cortex to form mature follicles. GCs continue to proliferate during this process. The thickness of the GC layer tends to increase in ph fc, reaching its thickest in follicles 6–8 mm in diameter. When the follicle is selected as dominant and enters the preovulatory sequence, the thickness of the GC layer gradually decreases with an increase in follicle volume until it forms a monolayer structure (Braw-Tal, 2002). Therefore, under the control of gonadotropins, the proliferation and differentiation of GCs result in different degrees of differentiation of GC populations (Woods and Johnson, 2005).

According to our sequencing data, a large number of DEGs related to cell proliferation were enriched, including *CCDC85C*, *EMP1*, and *RALY*, which is upregulated in phGC of high-laying hens.

Another class of DEGs is related to steroid hormone synthesis. Steroid hormones are mainly synthesized in the ovary and can regulate its growth and development, thereby affecting the reproductive function of females (Peluso, 2013; Peluso and Pru, 2014; Taraborrelli, 2015). There is a close relationship between follicle maturation and GC differentiation, and steroid hormones synthesized by GCs play crucial roles in follicular development and selection (Johnson, 2015; Kim and Johnson, 2018). We found that genes associated with steroidogenesis were upregulated in phGC of WL hens, whereas genes related to androgen production were upregulated in SF. These results are consistent with those of previous studies on high- and low-yielding hens (Brady et al., 2021). Among the genes upregulated in WL, *ADIPOQ* can stimulate progesterone secretion by regulating key steroidogenesis genes (*STAR*, *CYP11A1*, *FSHR*, and *LHR*) (Li et al., 2021). *CYP7B1* is present in reproductive organs and is an enzyme that metabolizes androgens (Stiles et al., 2009). *HSD3B1* is an important gene that catalyzes the conversion of pregnenolone to progesterone (Zhang et al., 2019). Among the genes upregulated in SF, *STC1* can inhibit FSH-induced progesterone production (Luo et al., 2004). *ADTRP* is an androgen-dependent regulatory protein (Patel et al., 2018). Notably, the expression of genes related to thyroid hormone synthesis were upregulated in SF. Studies have shown that thyroid hormone treatment can reduce FSH-stimulated estradiol in GCs of high-yield laying hens without reducing estradiol production in GCs of low-yield laying hens (Sechman et al., 2009; Brady et al., 2021). In addition, the upregulation of *DIO2* in low-yield layers may affect local thyroid hormone metabolism (Perfito et al., 2015). In addition, we observed the upregulation of oxytocin signaling in WL, including *CAMK2D* and *CD38*.

Finally, oocytes take up small molecule metabolites, such as energy substrates, nucleotides, and amino acids, from the surrounding GC medium through gap junctions (Gilchrist et al., 2004), to compensate for the lack of the low metabolic capacity of oocytes to take up small molecules (Sugiura et al., 2005). Through gap junctions within GCs, 85% of the metabolic needs of oocytes are met. Therefore, GCs play an important nutritional role in oocyte growth. GCs not only synthesize a variety of hormones but also generate a variety of growth factors and express their receptors, regulate the growth, differentiation, and maturation of theca cells and oocytes through gap junctions, and thereby regulate the development of follicles (Eppig, 2001). Our data showed that a large number of differential genes can be classified as factors related to cell growth, development, and information exchange. The upregulated genes of phGCs in WL are associated with various factors required for cell growth, including *ATPIF1*, a subunit of ATP synthase, which can limit the loss of ATP (Shah et al., 2012). *IGLL1* is an endometrial immune factor, and higher estrogen levels are associated with stronger immunity (Davoodi et al., 2016). *DNM1* mediates endocytosis of substances into oocytes. The upregulated genes in SF, including *STC1*, *GOLM1*, and *VCL*, have been reported to be related to ovarian development, promotion of Golgi membrane protein transfer, and cytoskeletal changes, respectively.

In addition, the expression of genes related to thyroid hormone synthesis, vascular smooth muscle contraction, and apoptosis pathways was upregulated in phGCs of SF. Elevated TSH levels lead to decreased estradiol and progesterone secretions (Sechman et al., 2009; Brady et al., 2021). The signaling pathway genes related to angiogenesis and vascular smooth muscle contraction were significantly upregulated. It is speculated that this may be related to the existence of a large amount of melanin outside the GC layer of SF, which prevents the inward transmission of substances and information; therefore, more abundant vascular tissue is needed to assist in transportation. Finally, what is special here is the enrichment of the melanin synthesis pathway, which corresponds to the special phenotype of SF. A large amount of melanin was distributed in the ovaries of SF, and the follicles were black as a whole, especially at ph fc (Figures 1A,B).

The poGCs were selected follicles with diameters of 9–40 mm. According to our sequencing data, a steroid genesis-related gene (*HSD11B2*) was upregulated in WL. Genes upregulated in low-laying hens, including *SD17B2*, are related to testosterone synthesis and secretion. *ZNF366* is a core inhibitor of estrogen receptors (Lopez-Garcia et al., 2006). Upregulation of genes related to thyroid hormone and insulin synthesis in low-laying hens, including the glucagon gene *GCG*. *IGF1*, is an insulin growth factor; *NR2F2* encodes the thyroid hormone gene; and *PTH1R* is a parathyroid hormone receptor. By comparing the expression levels of sex hormone synthesis-related genes and receptors in primary cultured high- and low-laying hens *in vitro*, we found that steroid synthesis-related genes and receptors were significantly higher in high-laying hens than in low-laying hens (Figure 5D). These *in vitro* experimental results are consistent with the sequencing results, indicating that the biological function of GCs in high-laying hens is stronger than that in low-laying hens.

In addition, the differential genes related to cell proliferation included *CCDC85C*, *MTFP1*, and *CHRDL1*, which were upregulated in high-laying hens. *BCAT1*, *FAM84B*, and *MYLK* were upregulated in low-laying hens. By comparing the proliferation ability of poGCs between high- and low-laying hens *in vitro*, we confirmed that the proliferation ability of GCs of high-laying hens was higher than that of low-laying hens (Figure 5C).

Finally, we identified various DEGs that are required for cell growth. The upregulated genes of high-laying hens included *TNC*, which has the function of cytohesin. *UGP2* regulates carbohydrate metabolism. *SCARB1* maintains ovarian cholesterol homeostasis and luteal steroid synthesis (Jimenez et al., 2010). The upregulated genes of low-laying hens included *MIIP*, *COL1A2*, and *CSRP2*, which have been shown to be migration proteins, cellular collagen, and cysteine proteins, respectively. In addition, we identified an important gene related to melanocyte production. *EDN3*, which was upregulated in SF, is involved in the migration and colonization of melanocytes. *KIT* is related to melanocyte formation. *HPGDS* is associated with melanoma formation. For the first time, we explored whether the presence of melanocytes affects the biological functions of GCs. Through the co-culture of ovarian melanocytes and GCs, and the overexpression of *EDN3* in GCs, we found that the ability of GCs to synthesize and secrete steroid hormones is affected (data not shown). Further in-depth research in this subject must be conducted in the future.

In the KEGG enrichment top 20 pathway, the upregulated genes of high-laying hens were enriched in signaling pathways, including fatty acid synthesis, metabolism, and degradation. Studies have shown that fatty acids are important enzymes in bovine GCs that promote cell proliferation, progesterone and estrogen secretion, and steroid synthesis (Plante-Dube et al., 2021). Genes related to the calcium signaling pathway are also very important. Studies have also shown that calcium can participate in the selection of follicles in laying ducks by activating the cAMP signaling pathway (Chen W. et al., 2020). The Wnt signaling pathway can promote the proliferation and differentiation of GCs and regulate the production of ovarian steroid hormones (Lapointe and Boerboom, 2011). Genes and signaling pathways upregulated in low-laying hens include the TNF signaling pathway, whose main function is to promote the biological effects of cell growth, differentiation, and apoptosis (Rodriguez et al., 2007). Compared with that in high-laying hens, we found that there were more apoptosis-related genes in low-laying hens. Genes related to the cGMP-PKG signaling pathway, insulin secretion signaling pathway, and epinephrine signaling pathway are upregulated in low-laying hens, which play an important role in maintaining the reproductive function of GCs (Jones et al., 1983; Adashi et al., 1985; Jaffe and Egbert, 2017). Similarly, we also found that the vascular smooth muscle contraction-signaling pathway and cytokine receptor interaction-signaling pathway are similar to those of phGCs. Thus, with the development of follicles, the size of the follicle increases, especially the increase in the deposition of yolk protein to oocytes. Furthermore, it is also possible that the presence of melanin increases the difficulty of GCs in transmitting information. Taken together, this study helps us understand the important reasons for the differences in egg production between WL and SF.

## Data Availability

All sequences were deposited in the National Center for Biotechnology Information (NCBI) and can be accessed in the Short Read Archive (SRA) under the accession number PRJNA856260.
