# Supplementary material for: Transcriptomic diversification of granulosa cells during follicular development between White Leghorn and Silky Fowl hens
Source: Front Genet. 2022 Jul 26;13:965414. doi: 10.3389/fgene.2022.965414 (PMC9360743; doi:10.3389/fgene.2022.965414)
Supplement: Supplementary file 1 [file Table1.DOCX]

**Table 1 The primers used to detect the targeted genes by qPCR**

| Genes | Primer sequence (5’-3’) | Annealing temperature | Size(bp) |
| --- | --- | --- | --- |
| WNT4 | F-CGAGCTGGACAAGTGTGGAT  R-GGACGTCGACAAAGGACTGT | 60 ^o^C | 125 |
| LHCGR | F-ACGAATCGCTGACACTCAAAC  R-CTCTCAGGGCATCGTTGTGT | 60 ^o^C | 138 |
| HSD11B2 | F-CACACCAATGGCACAGGTCTC  R-GTGCGGAAGTTGCCCAATG | 61 ^o^C | 98 |
| EDN3 | F-TCAACACCCCAGAGAGGACT  R-AGAGCACCGAAATGAAGGCT | 59 ^o^C | 116 |
| EDNRB2 | F-CCTCATTGCTCTGCCCATCA  R-GCCACTGCTCGATACCTGTC | 58 ^o^C | 159 |
| ADIPOQ | F- GAAGGGACGGCAAGG  R- TCACCCGTGTCCCCT | 56 ^o^C | 85 |
| CD38 | F-AGACAATTACAGGCCAGTCC  R-CATGTCCTTTTCAGTTCTGCAC | 57 ^o^C | 152 |
| STAR | F-CCATCAGCCAGGAGCTCAG  R-ATCTCGCTGAAGGGCTTCTC | 58 ^o^C | 124 |
| HSD3B1 | F-GCCAGAGGATCGTTCGCTTA  R-CATCTCGGATGTCCCCTTCC | 59 ^o^C | 153 |
| CYP11A1 | F-TACCGTGACTACCGCAACAA  R-AAAAAGTCCTGGCTCACCTGG | 60 ^o^C | 152 |
| FSHR | F-GAGCGAGGTCTACATACA  R-GCACAAGCCATAGTCA | 55 ^o^C | 281 |
| ER | F-TATTGATGATCGGCTTAGTCTGGC  R-CGAGCAGCAGTAGCCAGTAGCA | 63 ^o^C | 145 |
| GAPDH | F-TCGGAGTCAACGGATTTGGC  R-ACAGTGCCCTTGAAGTGTCC | 60 ^o^C | 163 |
