# Supplementary material for: Transcriptomic diversification of granulosa cells during follicular development between White Leghorn and Silky Fowl hens
Source: Front Genet. 2022 Jul 26;13:965414. doi: 10.3389/fgene.2022.965414 (PMC9360743; doi:10.3389/fgene.2022.965414)

**Supplementary information**

**Additional file 1** Egg production rate of White Leghorn and Silky Fowl.


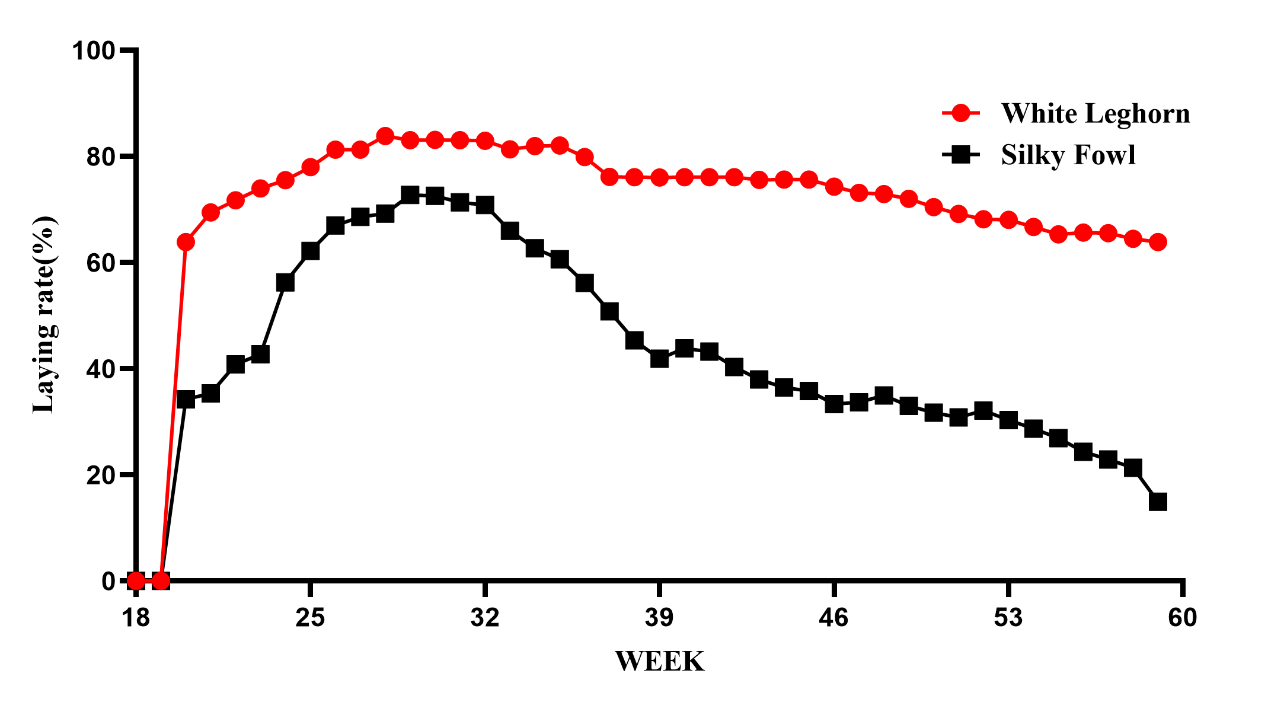


**Additional file 2** Gene ontology (GO) functional enrichment of genes differentially expressed between SF phGCs and WL phGCs. (The y-axis and x-axis indicate the percentage of genes in each cluster and the names of clusters respectively. )


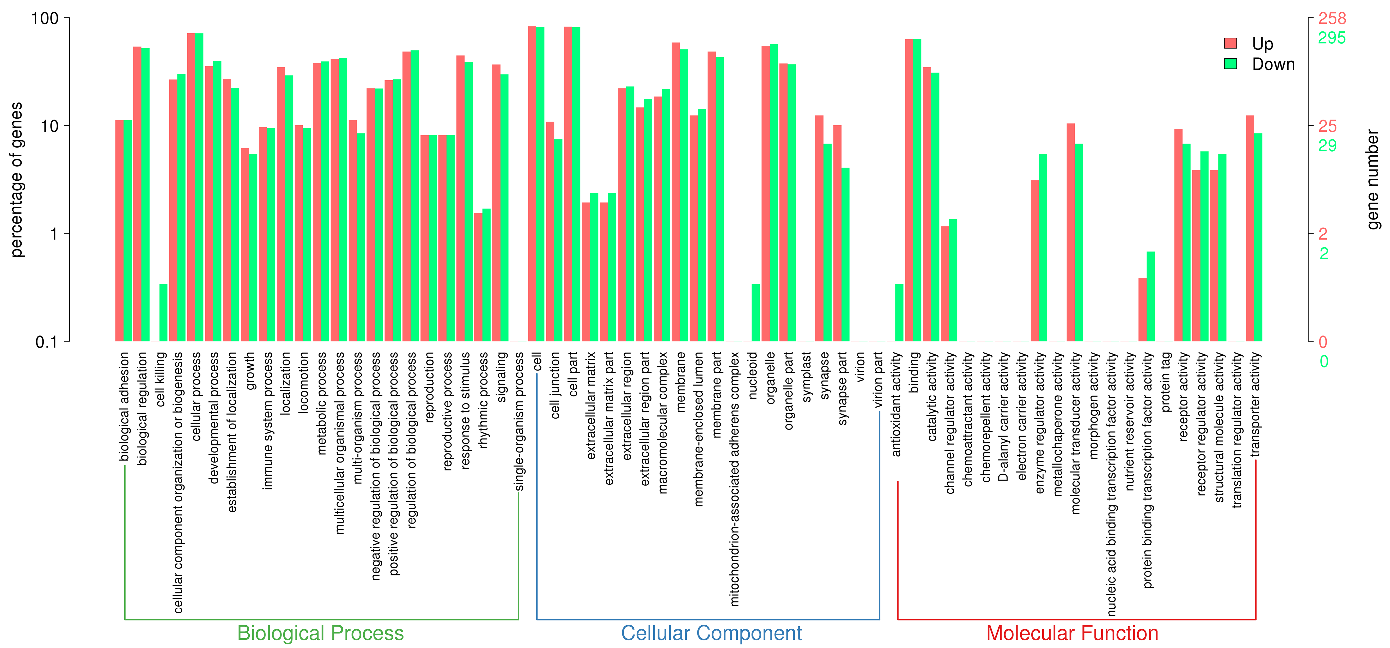


**Additional file 3** Gene ontology (GO) functional enrichment of genes differentially expressed between SF poGCs and WL poGCs.


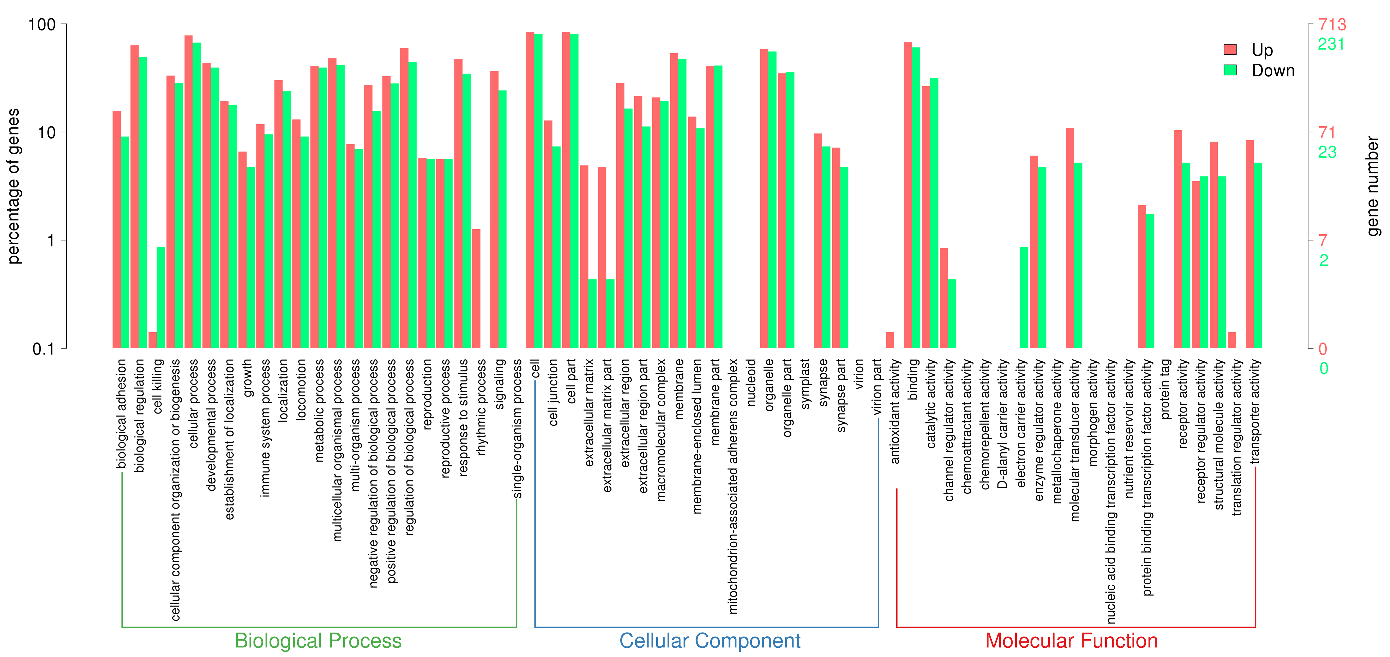

Supplement: Supplementary file 2 [file Table2.DOCX]
